# Decoding the Tackle: Using a Machine Learning Approach to Understand Direct Head Contact Events in Elite Women's Rugby

**DOI:** 10.1002/ejsc.70018

**Published:** 2025-08-03

**Authors:** Kathryn Dane, Ellen Rushe, Will Connors, Stephen W. West, Sharief Hendricks, Thomas Laurent, Ciaran Simms, Fiona Wilson, Anthony Ventresque

**Affiliations:** ^1^ Research Ireland Lero & School of Computer Science and Statistics Trinity College Dublin Dublin Ireland; ^2^ Discipline of Physiotherapy School of Medicine Trinity College Dublin Dublin Ireland; ^3^ School of Computing Dublin City University Dublin Ireland; ^4^ Department of Health Centre for Health and Injury & Illness Prevention in Sport University of Bath Bath UK; ^5^ UK Collaborating Centre on Injury and Illness Prevention in Sport University of Bath Bath UK; ^6^ Sport Injury Prevention Research Centre, Faculty of Kinesiology University of Calgary Calgary Canada; ^7^ Division of Physiological Sciences Department of Human Biology Faculty of Health Sciences University of Cape Town Rondebosch South Africa; ^8^ Carnegie Applied Rugby Research (CARR) Centre Carnegie School of Sport Leeds Beckett University Leeds UK; ^9^ Department of Mechanical Manufacturing and Biomedical Engineering & Centre for Biomedical Engineering Trinity College Dublin Dublin Ireland

**Keywords:** contact sport, injury prevention, sportswomen, technique

## Abstract

Concerns about the cumulative effects of head acceleration events in rugby are growing, but how tackle events lead to direct head contact in women's rugby remains underexplored. This cross‐sectional study aimed to develop and evaluate a machine learning model to identify characteristics associated with direct head contact and incorrect tackler head placement in elite women's rugby. Match situational and precontact technical characteristics (*n* = 31) from 1500 randomly selected tackle events were coded visually and retrospectively analyzed from the 2022–23 Women's Six Nations Championship. A machine learning model was developed and evaluated using a grid search with 5‐fold cross‐validations and F_1_ scores (i.e., a measure of predictive performance). The top modifiable characteristics associated with the target outcomes across 100 test sets were identified by mutual importance and decision tree modeling. The top modifiable characteristics linked to direct head contact to the tackler were incorrect head placement, coming to balance, and foot placement. Tackle direction, point of contact on the tackler, and multiplayer tackles were key characteristics for incorrect tackler head placement. Tackler drop height, front/oblique tackle direction, and multiplayer tackles were strongly associated with direct head contact to the ball‐carrier. Incorrect tackler head placement, the direction of tackle, tackler drop height, and multiplayer tackles are key characteristics in direct head contact events in elite women's rugby. Addressing these characteristics should be prioritized in contact training practices, education resources, and law enforcement to enhance player safety and direct head contact events in the women's game.

## Highlights

- A machine learning approach to modeling direct head contacts and incorrect head placement in elite women's rugby highlighted the inherent complexity of tackle events and the challenges in modeling characteristics associated with direct head contact events.
- Tackle technique coaching should focus on a tackler's ability to come to balance, tackler drop height, use correct head placement, and successfully defend front‐on/oblique and multiplayer tackles.
- The findings may support reinforcement or modification of current laws to address direct head contacts to the ball‐carrier.

## 1 Introduction

Rugby union (henceforth “rugby”) is a highly complex, fast‐paced game where success often depends on a team's defensive strategy, tackling proficiency and capacity (Hendricks et al. 2020; Scott et al. 2023). To contest the tackle as the ball‐carrier or tackler is a complex skill requiring appropriate physical attributes and precise decision‐making, timing, and coordination (Hendricks et al. 2020). During the tackle contest, players are exposed to head acceleration events (HAEs), defined as “an acceleration response of the head caused by an external short‐duration collision force applied directly to the head or indirectly via the body” (Woodward et al. 2024; Dane, West, Hendricks, et al. 2024). In the absence of head acceleration data, identifying direct head contacts is the best available proxy for potential HAE exposure (Woodward et al. 2024). In elite women's rugby match play, there are an average of 14 (95% CI 11–18) and 18 (95% CI 14–22) direct head contacts to tacklers and ball‐carriers per game, respectively (Dane, West, Hendricks, et al. 2024). The accumulation of HAEs has been postulated to have potential short‐ and long‐term health risks, including impacts on mental and physical health and associations with neurodegenerative disease (McKee et al. 2013; Montenigro et al. 2017; Stemper et al. 2019; Koerte et al. 2020).

Addressing the burden of injury from tackling requires identification of mechanisms that can be targeted to limit the exposure to direct head contact. Tackling technique has been identified as a potentially modifiable risk factor for direct head contacts in men's rugby union (Tucker et al. 2017; Hollander et al. 2021) and women's rugby league (Scantlebury et al. 2022). This research has informed the implementation of injury prevention measures such as law trials to lower the legal tackle height (World Rugby 2023a) and tackle safety programs (e.g., *Tackle Ready* World Rugby 2023b). However, these existing studies are almost exclusively based on men's rugby players (Dane et al. 2022). Androcentric approaches to head contact exposure mitigation may be misaligned to women's rugby players as they can commonly overlook differences in performance contexts (Dane et al. 2023), concussion injury rates (Shill et al. 2024), and mechanisms (Williams et al. 2022), as well as differences in physical, technical, and tactical aspects of match play between women's and men's rugby (Dane et al. 2022).

Statistical and machine learning (ML) approaches can identify feature association in complex datasets. For example, they can detect how specific tackle characteristics, such as direction of tackle or number of defenders involved, are statistically linked with the likelihood of direct head contact, making ML useful for exploring risk factors in rugby (Fan et al. 2014). ML approaches such as decision trees have been used to explore characteristics that increase the risk of tackler‐related concussions (Suzuki et al. 2021). In Japanese men's rugby, there was an 8‐fold higher odds of tackler concussion with incorrect head placement (Suzuki et al. 2021). Presently, the mechanisms for direct head contacts in women's rugby remain understudied, highlighting a critical knowledge gap in the literature. To identify characteristics associated with direct head contact events and incorrect tackler head placement in elite women's rugby, the present study aimed to develop and evaluate an ML model that incorporates ball‐carrier and tackler technical characteristics along with match situation variables.

## 2 Materials and Methods

### 2.1 Study Design

This retrospective cross‐sectional study formed part of a larger project investigating the technical characteristics of match tackle events in elite women's rugby (Dane, West, Hendricks, et al. 2024). Ethical approval was not required for commercially available match footage. This study used video analysis data from women's rugby players representing diverse ethnic backgrounds across France, England, Scotland, Ireland, Wales, and Italy, enhancing the generalizability of the findings across different populations. The reporting of the study follows the TRIPOD‐AI checklist for the reporting of prediction model studies (Table S1) (Collins et al. 2024).

### 2.2 Equity, Diversity, and Inclusion

Members of our research team included women and men, senior, mid, and early career academics with training in various disciplines (computer science, physiotherapy, engineering, sports science, epidemiology) from Europe and South Africa (global north and south).

### 2.3 Data Inputs: Tackle Technical Characteristics

A tackle was defined as “an event where one or more tacklers (player or players making the tackle) attempt to stop or impede the ball‐carrier (player carrying the ball) whether or not the ball‐carrier was brought to the ground” (Hendricks et al. 2020). Full methods have been reported by Dane, West, Hendricks, et al. 2024 but, in brief, a random sample of 1500 tackle events from all 15 games from the 2022–23 Women's Six Nations Championship was retrospectively analyzed. Video footage was coded using a technical framework adapted from a Rugby Union Video Analysis Consensus (RUVAC) (Hendricks et al. 2020), World Rugby's *Tackle Ready* (World Rugby 2023b), and feedback from an international group of women's rugby coaching stakeholders (*n* = 5) (Supporting Information S1: Table 2). The analysis demonstrated high intra‐ and inter‐rater reliability with *κ* values ranging from 0.90 to 1 on a subset of the data coded by other experts (Cohen 1960; Dane, West, Hendricks, et al. 2024). Baseline characteristics for all coded tackle events are provided by Dane, West, Hendricks, et al. 2024. For this study, 31 match situational and precontact characteristics preceding tackler/ball‐carrier head contact were included for analysis.

### 2.4 Data Target Outcomes: Direct Head Contact and Head Placement Data

**FIGURE 1:**
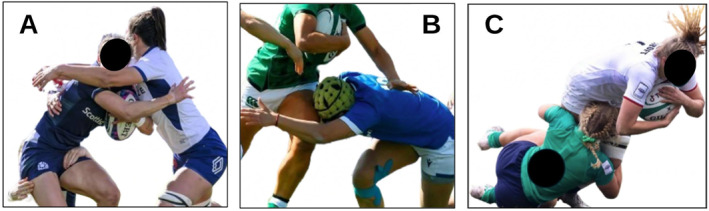
Visual description of (A) direct head contact to the ball‐carrier, (B) direct head contact to the tackler, (C) incorrect tackler head placement on contact.

Data collected from this larger project (Dane, West, Hendricks, et al. 2024) included tackle events resulting in incorrect tackler head placement (*n* = 231), direct contact to the head of the tackler (*n* = 78), or ball‐carrier (*n* = 94). These target outcomes were determined through visual inspection by a trained coder (*κ* = 0.99). “Incorrect tackler head placement” was defined as the tackler's head being in front of or on the incorrect side of the ball‐carrier during contact (Figure 1) (Hendricks et al. 2020). “Direct head contact” was defined as the initial contact between the head/face region of the ball‐carrier or tackler with another player's body part, equipment, or the ground during the initial contact phase, which may include unintended head‐to‐ground contact caused by loss of balance or tackle technique (Hendricks et al. 2020).

### 2.5 Machine Learning Model Development and Evaluation

**FIGURE 2:**
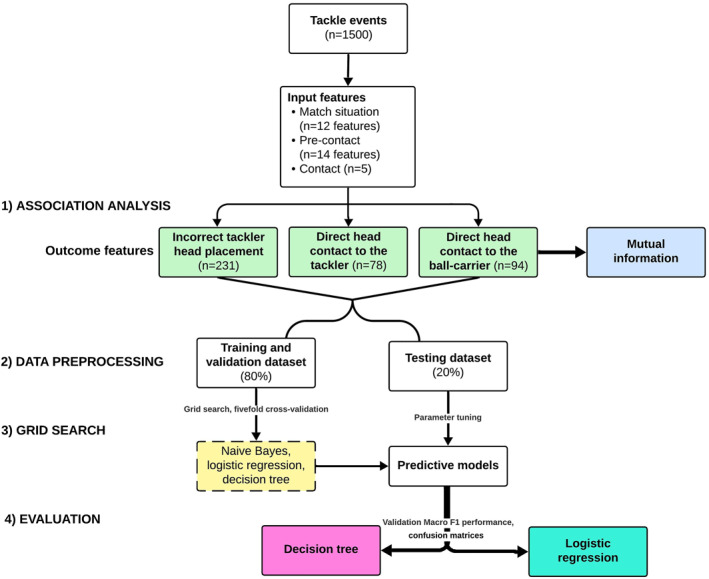
The workflow of data processing and ML modeling process.

An extensive glossary of key ML terms is provided in Supporting Information S1: Table 3. Model development and statistical analysis were performed in Python programming software (3.8.13). Our goal was to identify binary target outcomes: (1) tackler head placement (correct/incorrect) and direct head contact to (2) tackler and (3) ball‐carrier (yes/no) from the labeled input data. Discussions between rugby “field experts” (*n* = 2) and “data experts” (*n* = 3) were conducted to ensure a rigorous step‐by‐step procedure. Figure 2 shows the workflow of the ML model development and evaluation process. Scikit‐learn was used to train the predictive models (Pedregosa et al. 2011). We note that the target outcomes are naturally imbalanced within the dataset. We therefore evaluate both random oversampling and undersampling in our experiments to address this imbalance using the imbalanced‐learn Python package (LemaÎtre et al. 2017). The full code for this analysis is on GitHub.

### 2.6 Association Analysis

The pairwise association between the target outcomes and each characteristic were assessed independently to determine any strong associations between characteristic pairs. *Mutual information* (MI) was chosen to analyze this association, as it describes the amount of information one variable provides about another (Supporting Information S1: Figure 1) (Duncan 1970). MI quantifies how much knowing one variable (e.g., tackler body position) reduces uncertainty about another (e.g., occurrence of head contact) (Duncan 1970). We used this metric to quantify the importance of each characteristic to the target outcomes.

### 2.7 Data Preprocessing

We randomly split the dataset (*n* = 1500 tackles) into a training and validation set (80%) and a test set (20%). The training set was used to develop the prediction model, whereas the test set evaluated its performance. All features within the dataset are categorical and therefore to address class imbalance, we evaluated both random undersampling and oversampling (Kohavi 1995). A fivefold cross‐validation was used to prevent overfitting and assess the model's generalization (Kohavi 1995).

### 2.8 Grid Search

Seeking to balance the performance of this classification system with the interpretability (Gilpin et al. 2018) of the trained model, we limited analysis to naive Bayes, logistic regression, and decision tree models. To determine the best classification algorithm and corresponding parameters to use, we trained and validated each of the three models with a range of parameters applying a grid search and fivefold cross‐validation to optimize settings. The best hyperparameters found during cross‐validation were then used to train 100 models on the entire training set.

### 2.9 Decision Tree Modeling

We trained the decision tree model and then performed cross‐validation with hyperparameter optimization. To prevent the model fitting the data used in its training too closely, a phenomenon known as *overfitting* (Edouard et al. 2022), regularization was applied by limiting the maximum depth of trees to six during the grid search. To measure the optimal decision tree feature split, Gini impurity and information gain were included in the grid search (Hastie et al. 2009). Both are a measure of *impurity* (i.e., how well a particular decision will split the target feature into its composite classes).

### 2.10 Logistic Regression Modeling

A similar search strategy was performed for the logistic regression model. We applied regularization to the loss function of logistic regression models in the form of a penalty term. This term constrains the coefficients of the model by preventing them from growing too large and thus becoming overly influential. In this case, we evaluated with L1, L2, and elastic net (which combines L1 and L2) to determine the best regularization technique (Hastie et al. 2009). Additionally, we also experiment with different solvers and maximum numbers of iterations which are detailed in the paper's companion repository.

### 2.11 Evaluation

When evaluating the model, it is important to note that target outcomes are unevenly distributed (e.g., only 78 out of 1500 tackles resulted in direct tackler head contact). To address this, we used a macro‐averaged F_1_ score. This involved computing the F_1_ score (i.e., the harmonic mean of precision and recall) for each class separately and then averaging these scores to obtain a single performance metric (i.e., the macro‐averaged F_1_ score). Table 1 demonstrates how well each ML model performed in classifying target outcomes, compared to what would be expected by random chance. The decision tree models achieved the highest macro F_1_ score for tackler head placement and direct head contact. Logistic regression achieved the highest macro F_1_ score for ball‐carrier direct head contact (Table 1). However, though in all cases random oversampling led to the best model performance, this approach appeared to not be sufficient in addressing the natural class imbalance within the dataset and after evaluation using confusion matrices (Supporting Information S1: Figure 2), and mutual information was deemed a more suitable method for reporting ball‐carrier direct head contact results. The ML model performed better than random chance indicating moderate predictive power, but there is still room for optimization to reach higher performance.

**TABLE 1:** Results of grid search for target outcomes.

Target outcome	Best model	ML model validation macro F_1_ mean (std)	ML model test macro F_1_ mean (std)	Random chance macro F_1_ mean (std)
Tackler head placement	Decision tree	0.67 (0.03)	0.70 (< 0.0001)	0.44 (0.2)
Direct head contact to the tackler	Decision tree	0.65 (0.02)	0.64 (< 0.0001)	0.41 (0.3)
Direct head contact to the ball‐carrier	Logistic regression	0.61 (0.03)	0.55 (< 0.0001)	0.38 (0.3)

Abbreviation: Std, standard deviation.

## 3 Results

### 3.1 Mutual Information

Table 2 ranks the characteristics in terms of their importance for determining tackler head placement and direct head contacts in our model, as assessed using mutual information (MI). MI values range from zero (indicating no relationship) to one (indicating perfect dependence). There are no universally fixed cut‐off values for MI scores since they are context‐dependent (Edouard et al. 2022), but lower MI scores presented in Table 2 suggest weak relationships between these characteristics and the target outcomes.

**TABLE 2:** Mutual information (MI) and top three rankings for characteristics associated with tackler head placement, direct head contact to the tackler, and direct head contact to the ball‐carrier.

Target outcome	Tackler head placement		Direct head contact to the tackler		Direct head contact to the ball‐carrier	
Rank	Characteristic	MI score	Characteristic	MI score	Characteristic	MI score
1	Body region struck on tackler	0.0504	Tackler head placement	0.0504	Tackler drop height	0.0154
2	Direction of tackle	0.0347	Come to balance	0.0104	Multiplayer tackles	0.0151
3	Orientation of tackler	0.0230	Orientation of tackler	0.0055	Direction/orientation of tackle	0.0120

*Note:* The table shows the three most informative features per outcome. Results presented to 3 significant figures.

Abbreviation: MI, mutual information.

### 3.2 Direct Head Contacts to the Tackler

**FIGURE 3:**
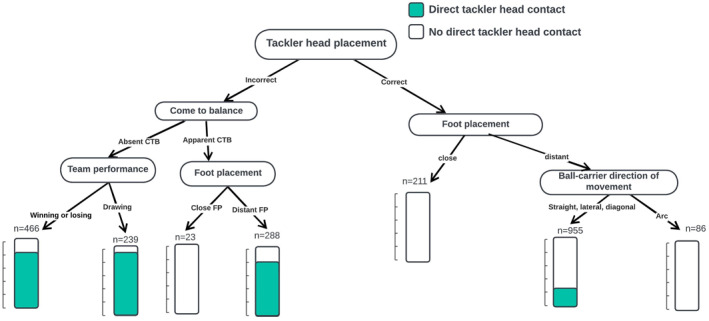
Decision tree of the characteristics associated with tackle events with a higher probability of direct head contact to the tackler at the time of contact. CTB: come to balance, FP: foot placement.

Figure 3 shows the decision tree model for characteristics associated with direct head contact to the tackler. Tackler head placement, located on the top of the tree, was the most important characteristic associated with tackler head contact during tackling. The frequency of tackles with direct head contact to the tackler increased when a tackler demonstrated incorrect head placement, an inability to come to balance (i.e., ability to adjust footwork and body position), and when defensive teams were drawing or when the tackler demonstrated distant foot placement. The direction of the ballcarrier's movement, whether running straight, laterally, or diagonally, was also associated with an increased likelihood of direct head contact.

### 3.3 Tackler Head Placement

**FIGURE 4:**
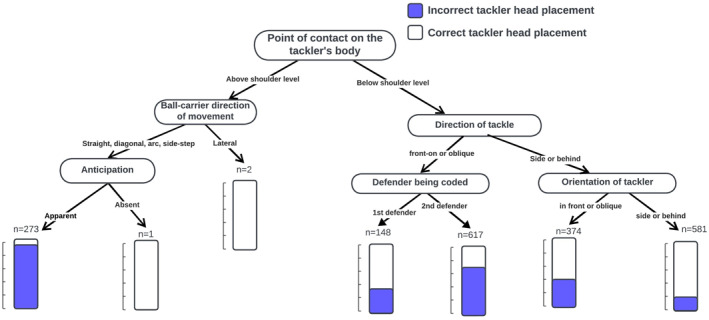
Decision tree of the characteristics associated with tackle events with a higher probability of incorrect tackler head placement at the time of contact.

The point of contact on the tackler's body (i.e., the specific body region of the tackler that is impacted by the ball‐carrier) was the most important characteristic associated with tackler head placement during contact (Figure 4). More specifically, tackles made above the level of the shoulder on the tackler showed incorrect head placement patterns. Additionally, tackler head placement varied depending on whether the ball‐carrier was running straight, diagonally, or in an arc. The likelihood of incorrect head placement increased for both first and second defenders in tackles made front‐on or oblique to the ball‐carrier, with a stronger association observed in second defenders.

### 3.4 Direct Head Contacts to the Ball‐Carrier

Logistic regression was identified as the best‐performing model for this outcome, and thus a decision tree was not presented. The characteristics with the strongest association with direct head contacts to the ball‐carrier were tackler drop height (i.e., correct timing of tackler lowering body height), multiplayer tackles, orientation of tackler pre‐contact, and the direction of tackle (Table 2).

## 4 Discussion

This study is the first to use ML models to identify the characteristics associated with direct head contact events and incorrect tackler head placement in elite women's rugby. Overall, these models showed moderate performance, but classified target outcomes better than random. Given the high number of tackle characteristics involved and the inherent complexity of the skill of tackling, achieving greater performance is difficult. However, our models identified several key characteristics influencing the probability of direct head contacts in elite women's rugby, with incorrect tackler head placement, front‐on and oblique tackle directions, the absence of tackler drop height, and multiplayer tackles emerging as the most important factors. Given current calls for reducing head acceleration exposure in rugby, these characteristics should be prioritized to reduce both the frequency and severity of direct head contact events in the women's game.

### 4.1 Direct Tackler Head Contacts

Our first important finding confirms the results of previous studies in women's (Spiegelhalter et al. 2023; Shill et al. 2024) and men's rugby (Hendricks et al. 2016; Tierney et al. 2018; Davidow et al. 2018; Sobue et al. 2018; Owen et al. 2024), that incorrect tackler head placement is strongly associated with direct tackler head contact during tackling. As reported in previous research (Dane, West, Hendricks, et al. 2024), several factors could contribute to tackler head placement during contact, including suboptimal decision‐making, insufficient contact exposure, and coaching in training (Dane et al. 2023). Tacklers must have appropriate physical and technical capacity to execute correct head placement repeatedly under fatigue (Hendricks et al. 2020). Existing contact safety resources should be evaluated and multilevel interventions (i.e., individual, organizational, institutional) are needed to support coaching practice in the evolving women's rugby infrastructure (Grindell et al. 2022; Hendricks et al. 2023).

Tacklers failing to “come to balance” were also strongly associated with direct tackler head contacts. Similarly, in male academy rugby players, the ability to change direction quickly and accelerate‐decelerate efficiently was associated with correct head placement in contact (den Hollander et al. 2023). Close foot placement and coming to balance are associated with superior tackle performance (World Rugby 2023b; Dane, West, Simms, et al. 2024). Therefore, given the associated benefits for injury prevention and performance (Dane, West, Simms, et al. 2024) of these techniques, existing tackle safety resources such as *Tackle Ready* (World Rugby 2023b) should be evaluated to support women's rugby coaches in targeting these precontact techniques.

In contrast to existing research on concussion risk in elite men's rugby union (Tucker et al. 2017; Cross et al. 2019) and women's rugby league (McLeod et al. 2023), this study found that neither tackler body position nor the body region struck on the ball‐carrier was strongly associated with direct head contacts to tacklers. These findings are further supported by work from Owen et al. (2025), who reported no significant differences in the risk of a head acceleration event (HAE) relative to body position or impact location in elite women's rugby union. This discrepancy may reflect differences in how features were defined and analyzed. Important predictors in our models—such as come to balance, foot placement, ball‐carrier direction of movement, team performance, and tackler head placement—were not included in earlier studies and may provide a more nuanced understanding of the mechanisms contributing to head contact in the women's game. Although direct head contacts represent only a subset of all HAEs in rugby tackling, they remain a crucial focus for injury prevention. Mechanisms of direct head contact may differ between genders, with women more frequently experiencing head‐to‐ground impacts (26.1%), which may not be as influenced by the body region struck or body position alone (Williams et al. 2022). Evaluations of tackle height law changes (World Rugby 2023a) have been conducted exclusively within men's rugby populations (van Tonder et al. 2023; Stokes et al. 2021). In a professional English men's rugby cohort, lowering the tackle height resulted in tacklers making contact with the ball‐carrier's head and neck 30% less often, although the tacklers themselves experienced a higher concussion rate rising from 6.9 to 13.2 concussions per 1000 hours of play (Stokes et al. 2021). Nonetheless, tackle height remains an important focus for reducing direct head‐to‐head contacts and sub‐concussive HAEs. Findings from this study suggest that executing proper tackling techniques—such as correct head placement, balance, foot positioning, and drop in height—may be more critical for reducing direct head contacts to tacklers than tackler body position alone. However, the observed execution of recommended *Tackle Ready* (World Rugby 2023b) techniques in this cohort only averages 47% with 0.2% (*n* = 3/1500 tackles) of the coded tackles demonstrating all 22 coded recommended techniques (Dane, West, Hendricks, et al. 2024). Low adoption of recommended techniques is multifactorial (Stodter and Dane 2024); therefore, multilevel engagement (e.g., players, coaches, researchers, match‐officials) is needed to understand and improve implementation to maximize adoption.

### 4.2 Tackler Head Placement

The point of contact on the tackler was the most important characteristic associated with tackler head placement during contact. This highlights that head placement is a critical component of overall tackling technique. Regardless of the correctness of other technical aspects, poor head placement alone indicates poor technique (Davidow et al. 2018; Hollander et al. 2021). This finding supports current coaching education initiatives focused on correct tackler head placement to reduce direct head contact exposure in elite women's rugby. Although previous research has identified precontact head positioning influences head placement (Suzuki et al. 2021; Shill et al. 2024), our modeling suggests that this relationship may be more closely linked with insufficient tackler “drop height” during the approach. In other words, failing to lower body height adequately, precontact may increase the likelihood of incorrect head placement.

The direction of tackle and orientation of the tackler to the ballcarrier were the only characteristics identified across all data target outcomes, underscoring their central role in influencing head impact risk and injury prevention strategies in women's rugby. Mirroring findings in elite men's rugby (Tucker et al. 2017), front‐on or oblique tackles demonstrated a stronger association with incorrect tackler head placement and direct head contacts to tacklers and ball‐carriers. Importantly, this association does not appear to be a consequence of exposure frequency: In our dataset, front‐on (5.2%) and oblique (29%) tackles were less common than tackles from behind (36%) or side‐on (31%). Despite their lower frequency, front‐on and oblique tackles were disproportionately linked with elevated head contact risk, suggesting that it is the technical demands of these tackle types—rather than their prevalence—that heighten the risk. This pattern may also reflect game dynamics: When tackling from behind or the side, the tackler's head is more likely to be positioned behind the ball‐carrier, naturally reducing the likelihood of direct contact. Additionally, factors such as tackling with the incorrect shoulder or late carrier movement may further influence head contact risk, highlighting the complexity of tackle dynamics. It is also important to consider that the sport's nature often limits the tackler's ability to alter tackle direction, resulting in a high proportion of front‐on and oblique tackles and, consequently, head contacts from these angles. Due to superior performance outcomes (Dane, West, Simms, et al. 2024), existing coaching resources consistently reinforce the messaging of front‐on/oblique tackles to “get square” to limit the ballcarrier's momentum and create dominant tackles (World Rugby 2023b). Recognizing the inherent tension between performance effectiveness and player safety (Dane et al. 2023), rather than abandoning front‐on/oblique tackles, coaching should focus on sufficient technical preparation prioritizing correct head placement and body positioning to safely execute those tackle types.

### 4.3 Direct Ball‐Carrier Head Contacts

Under law 9.11 and its Head Contact Process, World Rugby enforces sanctions for high‐risk tackle behaviors that result in head or neck contact, including high tackles (World Rugby 2024). However, the observed prevalence for direct ball‐carrier head contacts (18 per match) combined with the finding that only 8 penalties per match were awarded on average for all possible tackle infringements suggests that sanctioning of this law violation needs stricter enforcement.

Tackler “drop height” emerged as the most important factor for direct head contact with the ball‐carrier. Sudden, late changes in the ball‐carrier's body height can disrupt a tackler's ability to execute this movement effectively. Law trials by the Fédération Française de Rugby (FFR) that prohibit late changes in the ball‐carrier's body height (England Rugby 2023) may help mitigate this issue by preserving the tackler's opportunity to safely drop their height, potentially enhancing the tackler's ability to avoid direct head impacts to ball‐carriers. Although not previously examined, the tackler's capacity to drop into a lower body position depends on sufficient leg strength, flexibility, and swift perception‐action capabilities in response to player movements. Addressing late changes in ball‐carrier height through law changes, alongside optimizing contact preparation in training (Hendricks et al. 2018), could be pivotal for reducing head contacts in women's rugby.

Multi‐defender tackles were also associated with direct head contacts to the ball‐carrier. In theory, although potentially effective in limiting ballcarrier momentum, multiplayer involvement in a single tackle limits the contact points available on a ball‐carrier's body, therefore bringing tacklers into unintended proximity with the ball‐carrier's head. The FFR has also trialed law changes to prohibit simultaneous two‐defender tackles (England Rugby 2023). Given the present findings and those of female varsity rugby (Williams et al. 2022), there is support to restrict the number of defenders in the tackle to limit the potential for direct head contacts. However, with multiplayer tackles accounting for 48% of elite women's rugby tackles (Dane, West, Hendricks, et al. 2024), sufficient training exposure to these scenarios—along with focused coaching on technique, decision‐making, and tactical roles—is essential to prepare players for these match demands.

### 4.4 Uniformity in Mutual Information

The lower MI scores reflect the complex, dynamic nature of rugby tackles where multiple characteristics interact simultaneously (Duncan 1970; Gilpin et al. 2018). Additionally, the relative uniformity in MI could be attributed to several factors, including but not limited to (1) low dependency, that is, the characteristics provide little insights about the target outcome or (2) redundancy, that is, multiple characteristics overlap, providing the same information about the target outcome. Overall, the relative uniformity in MI scores suggests that individual characteristics alone cannot fully predict direct head contacts (Passos et al. 2008). Future research should consider further modeling approaches that can capture the co‐occurrence and temporal ordering of tackle characteristics. This could provide more meaningful insights into how combinations of factors—not just isolated features—contribute to injury risk in the tackle.

### 4.5 Clinical Implications

Clinical implications of these findings center on the need for the targeted prevention of direct head contacts in women's rugby through specific tackle technique training. Players with proper balance before contact, appropriate drop height, and correct head placement during tackles may have reduced risk of head contact incidents, particularly during front‐on/oblique and multiplayer tackle situations. These findings may promote targeted prevention strategies and education focusing on these specific technical elements to help reduce the risk of head injuries.

## 5 Limitations

The deterministic approach to video analysis applied in the present study may not fully capture the dynamic complexity of tackling. Model interpretability does not imply causality for head injuries (Leckey et al. 2024). Future studies should assess whether characteristics linked to direct head contact also correlate with head impact assessments and concussions (Sobue et al. 2018; Shill et al. 2024). Given the challenges of manually labeling tackle technical characteristics, pose estimation offers an opportunity to improve the accuracy and depth of tackle technique analysis by automating this process (Connors et al. 2024). Secondary head‐to‐ground contacts represent an important area for future research (Williams et al. 2022) and incorporating instrumented mouthguard data could help quantify both direct and indirect HAEs (Woodward et al. 2024; Allan et al. 2024). Future work should also apply this machine learning approach to men's rugby, enabling cross‐sex comparisons and testing the generalizabilty of findings beyond women's rugby. Research should also expand to community settings, age categories, and southern hemisphere contexts.

## 6 Conclusion

This is the first study to develop and evaluate an ML model to identify characteristics associated with direct head contact and incorrect tackler head placement from coded video data in elite women's rugby. Findings support the need for greater attention to coach education and the development of tackle coaching strategies tailored to the specific demands observed in the women's game—particularly focusing on a tackler's ability to come to balance, drop height, use correct head placement, and safely execute front‐on/oblique and multiplayer tackles. Drop height and multiplayer tackle considerations could inform reinforcement or modification of laws to address direct head contacts to the ball‐carrier. The ML approach provides a foundation for future research to design and evaluate the effectiveness of tackle safety interventions in women's rugby, integrating both clinical outcomes and wearable technology.

## Author Contributions

The study was designed by K.D., F.W., C.S. and A.V. Data collection was carried out by K.D., C.O.B. and C.S. Data analysis was carried out by K.D., E.R. and T.L. The first draft of the manuscript was prepared by K.D., T.L. and E.R. All authors contributed to revisions of the manuscript and approved the submitted version.

## Ethics Statement

The authors have nothing to report.

## Conflicts of Interest

SH is an Associate Editor and Social Media Editor of the European Journal of Sport Sciences.

## Supporting information

Supporting Information S1

Table S1

## Data Availability

The network and source code for the preprocessing steps and data analysis pipeline performed for the study are available at https://github.com/EllenRushe/DecodingTheTackle. The original data supporting the findings of this study are available from the corresponding author upon reasonable request.
