# Supplementary material for: Decoding the Tackle: Using a Machine Learning Approach to Understand Direct Head Contact Events in Elite Women's Rugby
Source: Eur J Sport Sci. 2025 Aug 3;25(8):e70018. doi: 10.1002/ejsc.70018 (PMC12318822; doi:10.1002/ejsc.70018)
Supplement: Supplementary file 1 — Supporting Information S1 [file EJSC-25-e70018-s002.pdf]

Supplementary Table 2- Tackle descriptors and definitions

General operational variables	Description
**Tackle event**	an event where one or more tacklers attempt to stop or impede the ball-carrier whether or not the ball-carrier was brought to ground.
**Missed tackle**	The BC successfully penetrates the attempted tackle and continues to advance
**Completed tackle**	When a tackle break does not occur, and either player goes to ground or the ball-carrier is held up and cannot progress further.
**Defender/Tackler**	Player/s involved in the tackle seeking to regain possession of the ball from a ball carrier
**Attacker**	Player/s involved in the tackle in possession of the ball
**Ball-carrier**	A player in possession of the ball
**CONTEXTUAL VARIABLES**	
**1. Previous phase or set piece**	The phase or set piece preceding the tackle event.<br>Ruck<br>Lineout<br>Scrum<br>Restart
**2. Pass number**	The number of passes from the previous phase or set piece until the tackle event
**3. Match period**	1st quarter: 0-20 minutes<br>2nd quarter: 20-40 minutes<br>3rd quarter: 40-60 minutes<br>4th quarter: 60-80 minutes
**4. Defensive direction**<br>a) Lateral<br>b) Backwards<br>c) Forwards<br>d) No direction	Direction of movement executed by the defensive line (5 seconds preceding the tackle event)<br>a) Approaching the BC laterally<br>b) Retreating from the BC<br>c) Approaching the BC front-on<br>d) No identifiable movement direction (not moving lateral, backwards or forwards)
**5. Number of defenders**	1,2,3,4- how many defenders were involved in the tackle event?
**6. Defender being coded**	1,2,3,4 e.g. 1= primary tackler, 2=secondary tackler
**7. Tackle sequence**<br>a) One on one<br>b) Sequential<br>c) Simultaneous<br>d) Attacking sequential	The sequence of contact made by either by one or more tacklers (at and immediately after the initial point of contact)<br>a) One tackler contacts BC<br>b) One tackler contacts BC, followed by a second defender joining the contact situation c) Two tacklers contact BC at the same time<br>d) Two attackers contact one tackler after each other
**8. Positional grouping of the tackler**	a) Backs: position numbers 9-15<br>b) Forwards: position numbers 1-8
**9. Positional grouping of the ballcarrier**	a) Backs: position numbers 9-15<br>b) Forwards: position numbers 1-8
**10. Defending team status**<br>a) Winning<br>b) Losing<br>c) Drawing	a) defending team was winning at the time of the tackle event<br>b) defending team was losing at the time of the tackle event<br>c) defending and attacking teams were drawing at the time of the tackle event
**PRECONTACT VARIABLES**	
**11. Distance of the BC from tackler at ball reception**<br>a) Near	Distance between the tackler and BC before the tackle (0.5 seconds preceding the tackle event)<br>a) Less than 2m of the tackler<br>b) Between 2-4m of the tackler

b) Moderate<br>c) Distant	c) Greater than 4m from the tackler
**12. Anticipation of BC**<br>a) Apparent<br>b) Absent	Evidence that tackler is attuned and aware of BC and impending contact (0.5 seconds preceding the tackle event)<br>a) tackler was aware of impending contact<br>b) tackler was unaware of impending contact
**13. Come to balance***<br>a) Apparent<br>b) Absent	Does the tackler adjust body position and footwork to BC change of direction? (yes/no)
**14. Body position of BC**<br>a) Upright<br>b) Medium<br>c) Low	Position of the BC's upper body relative to their lower body i.e. degree of upper body flexion/extension (0.5 seconds preceding the tackle event)<br>a) BC displayed high body height with knees and hips extended<br>b) BC displayed moderate flexion at knees and hips<br>c) BC displayed low body height
**15. Body position of tackler**<br>a) Upright<br>b) Medium<br>c) Low	Position of the tacklers upper body relative to their lower body i.e. degree of upper body flexion/extension (0.5 seconds preceding the tackle event)<br>a) tackler displayed high body height with knees and/or hips extended<br>b) tackler displayed moderate flexion at knees and/or hips.<br>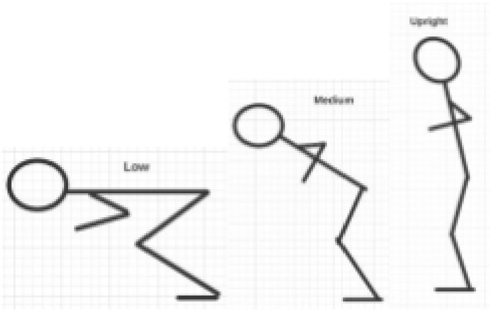<br><br>c) tackler displayed low body height
**16. Drop height***<br>a) Apparent<br>b) Absent	Does the tackler stay big and drop height appropriately at the correct time? (yes/no)
**17. Dominant contact angle***	Does the tackler demonstrate a straight line posture through hips, spine, head up, chin off chest and eyes on target? (yes/no)<br>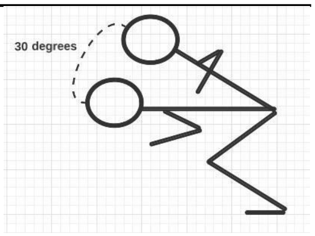
**18. Head position of tackler**<br>a) Up and forward<br>b) Away<br>c) Down	Position of the tackler's head in relation to the position of the BC<br>a) Gaze focused on BC<br>b) Gaze away from BC<br>c) Gaze towards the ground
**19. Arm position of tackler**<br>a) Hands above shoulders<br>b) Hands dropped<br>c) Boxer stance/claws up elbows in*	Position of the tackler's arms before the tackle (0.5 seconds preceding the tackle event)<br>a) Hands of the tackler are raised above their shoulders<br>b) Hands of the tackler are dropped below the level of their elbows<br>c) tackler's elbows are bent with at least one hand raised above the level of their elbow
**20. Foot placement close to BC***<br>a) Apparent<br>b) Absent	Does the tackler get their foot as close as possible to the BC? (foot placement in line with BC centre of mass e.g. point on ground directly below BC's hips) (yes/no)
**21. Shoulders in front of hips***	Are the tacklers shoulders in front of hips in a loaded body position? (yes/no)

**22. Speed of tackler**<br>a) Fast (maximal)<br>b) Moderate (sub-maximal)<br>c) Slow (static)	Estimation of the tackler's running speed (subjective assessment) (0.5 seconds preceding the tackle event)<br>a) Running or sprinting (purposeful running with maximal effort, with high knee lift)<br>b) Jogging (non-purposeful slow running with low knee lift)<br>c) Stationary or walking (no visible knee lift no visible knee lift or rapid foot movement)
**23. Speed of BC**<br>a) Fast (maximal)<br>b) Moderate (sub-maximal)<br>c) Slow (static)	Estimation of the BC's running speed (subjective assessment) (0.5 seconds preceding the tackle event)<br>a) Running or sprinting (purposeful running with maximal effort, with high knee lift)<br>b) Jogging (non-purposeful slow running with low knee lift)<br>c) Stationary or walking (no visible knee lift or rapid foot movement)
**24. Direction of movement of BC**<br>a) Straight<br>b) Side Step	Movement of the BC before the tackle (0.5 seconds preceding the tackle event)<br>a) BC ran straight at the defence<br>b) BC performed an evasive step initiated by either leg

c) Arcing run<br>d) Lateral run<br>e) Diagonal run	c) BC performed arcing run<br>d) BC performed a run from touchline to touchline<br>e) BC runs at an angle, instead of straight at the tackler
**25. Orientation of tackler in relation to BC***<br>a) In-front<br>b) Side<br>c) Oblique<br>d) Behind	Position of the tackler in relation to the BC (0.5 seconds preceding the tackle event)<br>a) Tackler and BC moving head on toward each other<br>b) Tackler moving in from the BC's side<br>c) Tackler moving towards BC at an angle somewhere between the front-on and side-on positions. d) Tackler chasing BC toward own try-line
	**CONTACT VARIABLES**
**26. Body region of the tackler struck**<br>a) Lower leg<br>b) Hip<br>c) Upper leg<br>d) Torso<br>e) Shoulder<br>f) Arm<br>g) Head and neck	The initial anatomical location contacted on the tackler by the BC (at and immediately after the initial point of contact)<br>a) Area below the knee<br>b) On the shorts line (belly button to bottom of shorts)<br>c) Area between bottom of shorts and knees<br>d) Above the BC's belly button to the armpit<br>e) Armpit to the shoulder<br>f) Contact only to the arm initially<br>g) Above the shoulder (contact with the head/neck)
**27. Body region of the BC struck***<br>a) Lower leg<br>b) Hip<br>c) Upper leg<br>d) Torso<br>e) Shoulder<br>f) Arm<br>g) Head and neck<br>h) Ball	The initial anatomical location contacted on the BC by the tackler (at and immediately after the initial point of contact)<br>a) Area below the knee<br>b) On the shorts line (belly button to bottom of shorts)<br>c) Area between bottom of shorts and knees<br>d) Above the BC's belly button to the armpit<br>e) Armpit to the shoulder<br>f) Contact only to the arm initially<br>g) Above the shoulder (contact with the head/neck)<br>h) Contact to the ball initially
**28. Type of tackle**<br>a) Arm tackle<br>b) Jersey tackle<br>c) Smother tackle<br>d) Shoulder tackle<br>e) Tap tackle	The type of tackle technique executed by the tackler (at and immediately after the initial point of contact) a) Tackler impedes BC with the upper limbs<br>b) Tackler holds BC's jersey<br>c) Tackler uses chest and wraps both arms around BC<br>d) Tackler makes initial contact with their shoulder and wraps the BC with their arm<br>e) Tackler trips BC with hand on lower limb below the knee

**29. Direction of tackle**<br>a) Front-on<br>b) Side-on<br>c) Oblique<br>d) Behind	The direction from where the tackler makes contact with the BC (at and immediately after the initial point of contact)<br>a) Tackler makes initial contact head-on with BC<br>b) Tackler makes initial contact with the BC's side<br>c) Tackler makes initial contact with BC at an angle<br>d) Tackler makes initial contact with BC from behind
**30. Head placement of the tackler**<br>a) Above<br>b) Beside<br>c) In-front<br>d) Behind	The position of the tackler's head in relation to the BC (at and immediately after the initial point of contact) a) Head higher than BC's body during contact<br>b) Head next to BC's body during contact<br>c) Head in front of BC's body during contact<br>d) Head at the back of BC's body during contact
**31. Ear to body ***	Does the tackler's ear stay in contact with BC's body? (arrowhead- extra clamp with head) yes/no
**32. Arm wrap and clamp***<br>a) No attempt to wrap<br>b) Wrap<br>c) Failed wrap	Does the tackler use arms to wrap and clamp the BC?<br>a) Tackler makes no attempt to wrap BC<br>b) Tackler wraps BC<br>c) Tackler attempts to wrap BC but loses grip
**33. BC fend**<br>a) Absent<br>b) Moderate<br>c) Strong	Arm movement of the BC to repel the efforts of the tackler(s) (at and immediately after the initial point of contact)<br>a) BC provided no fend<br>b) BC provided a light to moderate fend (e.g. swat or slap technique)<br>c) BC provided strong fend (e.g. push technique)
**34. Tackler shoulder usage**<br>a) Shoulder usage<br>b) No shoulder usage	Use of a shoulder by the tackler after initial contact (0.5 seconds after the tackle event)<br>a) Tackler uses shoulder during or after initial contact is made<br>b) No shoulder usage from tackler after initial contact is made
**35. Shoulder usage active or passive or N/A?***	If shoulder was used, was it active or passive<br>Active: first contact with the tacklers shoulder, and the tackler drives or attempts to drive the BC backwards<br>Passive: first contact with the tacklers shoulder, and no attempt to drive the BC backwards N/A
**36. Roll over front foot***	Does the tackler weight shift to the front foot? (yes/no)
**37. Studs in the grass***	Are the tacklers studs in the grass, no dragging toes? (yes/no)
	**POSTCONTACT VARIABLES**
**38. Tackler leg drive***<br>a) Absent<br>b) Moderate<br>c) Strong	Leg drive executed by the tackler after initial contact (0.5 seconds after the tackle event) a) No leg drive<br>b) Moderate knee movement, with no high lift<br>c) High, rapid knee lift
**39. BC leg drive**<br>a) Absent<br>b) Moderate<br>c) Strong	Leg drive executed by the BC after initial contact (0.5 seconds after the tackle event) a) No leg drive<br>b) Moderate knee movement, with no high lift<br>c) High, rapid knee lift
**40. Post tackle effort***<br>a) Bounce<br>b) Jackal/poach<br>c) Barge/disrupt attempt<br>d) Disrupt success<br>e) LOG<br>f) Tackler trapped	First effort made by the tackler (0.5 seconds after the tackle event)<br>a) Bounce: When the tackler gets up from the ground and returns to the defensive line before the ball is played from the ruck.<br>b) Jackal/Poach: Tackler picks the ball up from a tackled player while remaining on feet, from arriving on the correct side of the tackle, all before the ruck is formed.<br>c) Barge/Disrupt attempt: Tackler gets up from ground and tries to disrupt attacking ruck- no change to ruck ball.<br>d) Disrupt success: Tackler gets up from ground and tries to disrupt attacking ruck- ruck ball is slowed.<br>e) LOG: Tackler lying on the ground when attacking team plays ball<br>f) Trapped: Tackler is too slow to roll away, cannot escape ruck – may concede penalty for not rolling away

**41. Finish on top/dominance***<br>a) Tackler dominance<br>b) BC dominance	Who dominated the contact:<br>a) Tackler finishes on top of BC<br>b) BC finishes on top of tackler
**PERFORMANCE OUTCOMES**	
**42. Which player achieves contact territorial/gainline dominance?** a) BC<br>b) Tackler<br>c) No change	The direction of progression the tackler and ball-carrier made (as a single unit) towards the opposition try-line from the previous ruck to the point where both players went to ground (completed tackle) or when a maul is formed. a) Tackle finishes closer to defensive try-line<br>b) Tackle finishes closer to attacking try-line<br>c) Tackle finishes in same place as previous ruck
**43. Tackle result**<br>a) Offload<br>b) Tackle break<br>c) Ruck formed<br>d) Turnover won<br>e) Tackler penalty conceded (pen -)<br>f) BC penalty conceded (pen +)<br>g) Other (try scored)	Overall outcome after the tackle (5 seconds post the tackle event)<br>a) Offload: ball-carrier is able to pass the ball to a teammate during the tackle.<br>b) Tackle break: the BC successfully penetrates the attempted tackle and continues to advance.<br>c) Ruck formed: a phase of play whereby one or more players from each team, who are on their feet, in physical contact, close around the ball on the ground.<br>d) Turnover won: the attacking team is unable to advance through an offload or tackle break, or form a ruck, and lose the ball either through an infringement or error. e) Tackler concedes penalty.<br>f) BC concedes penalty.<br>g)BC scores try.

*- relevant to TackleReady performance criteria (World Rugby, 2022). LOG- lying on ground, BC- ball-carrier, pen- penalty against defending team, pen+ penalty against attacking team.

**Supplementary Table 3** Terminology and definitions	
**Term**	**Definition**
**Machine Learning (ML)**	A subset of artificial intelligence that involves the development of algorithms that allow a system to learn from data and improve performance without explicit programming (Mitchell, 1997).
**Binary Target Outcomes**	The outcomes of interest, specifically tackler head placement (correct/incorrect) and direct head contact (yes/no) (Mitchell, 1997).
**Scikit-learn**	A tool used in Python programming to help build machine learning models and analyse data (Pedregosa et al., 2011).
**Mutual Information (MI)**	A way to measure how much knowing one variable helps predict another (Duncan, 1970).
**Entropy**	A measure of unpredictability or disorder in a system (Shannon, 1948).
**Training Set**	The portion of the dataset (typically 80%) used to train a model to recognise patterns (Edouard, Verhagen, & Navarro, 2022).
**Test Set**	The part of the data used to test how well the model performs on new, unseen data (Edouard, Verhagen, & Navarro, 2022).
**Fivefold Cross-Validation**	A technique for testing a model's performance by splitting the data into five parts, using four to train and one to test, and repeating the process five times (Kohavi, 1995).
**Grid Search**	A method of finding the best settings for a model by testing multiple combinations of parameters (Hutter, Hoos, & Leyton-Brown, 2011).
**Hyperparameters**	Settings chosen before training a model, such as how fast it learns or how complex it can be, which are adjusted to improve the model's performance (Hutter, Hoos, & Leyton-Brown, 2011).
**Decision Tree Model**	A model that makes predictions by splitting data into branches based on certain features, much like following a decision tree.
**Information Gain**	A measure of how much a feature helps in dividing data into useful categories (Duncan, 1970).
**Overfitting**	A problem where a model learns the noise or random fluctuations in the training data rather than the underlying pattern, leading to poor generalisation on new data (Edouard, Verhagen, & Navarro, 2022).
**Regularisation**	A method to prevent overfitting by discouraging overly complex models (Hastie, 2009).
**L1 Regularisation (Lasso)**	A technique that simplifies a model by making some features less important.
**L2 Regularisation (Ridge)**	A technique that reduces the size of the models coefficients
**ElasticNet Regularisation**	A combination of L1 and L2 regularization that balances the benefits of both techniques.
**Macro-Averaged F1-Score**	A way to evaluate a model by calculating its predictive performance for each class and then averaging those results to give equal importance to each class (Edouard, Verhagen, & Navarro, 2022).
**Confusion Matrix**	A tool to assess the performance of a model by showing how many predictions were correct or wrong for each class.
**Predictive Performance**	How well a model is able to make accurate predictions on new, unseen data.
**Class Imbalance**	When one class in a dataset is much more common than another, which can lead to biased models that favour the more common class.
**Precision**	The proportion of true positive predictions out of all positive predictions made by the model (Powers, 2011).
**Recall**	The proportion of true positive predictions out of all actual positive instances in the data (Powers, 2011).

References

1. Mitchell, T.M. (1997). “Machine Learning”. McGraw-Hill.
2. Pedregosa, F., Varoquaux, G., Gramfort, A., Michel, V., Thirion, B., Grisel, O., ... & Duchesnay, É. (2011). “Scikit-learn: Machine learning in Python.” *the Journal of machine Learning research*, *12*, 2825-2830.
3. Duncan, T.E. (1970). “On the calculation of mutual information.” *SIAM J Appl Math*; 19(1): 215-220.
4. Shannon, C.E. (1948). “*A Mathematical Theory of Communication*.” *Bell Syst Tech J*; 27(3): 379-423.
5. Edouard, P., Verhagen, E., & Navarro, L. (2022). “Machine learning analyses can be of interest to estimate the risk of injury in sports injury and rehabilitation.” *Annals of physical and rehabilitation medicine*, *65*(4), 101431.
6. Kohavi, R. (1995). “A study of cross-validation and bootstrap for accuracy estimation and model selection.” In *Ijcai* (Vol. 14, No. 2, pp. 1137-1145).
7. Hutter, F., Hoos, H.H., Leyton-Brown, K. (2011). “Sequential Model-Based Optimization for General Algorithm Configuration.” *Proceedings of the 5th International Conference on Learning and Intelligent Optimization*; 355-372.
8. Hastie, T. (2009). “The elements of statistical learning: data mining, inference, and prediction.”
9. Powers, D.M. (2011). “Evaluation: From Precision, Recall and F-Score to ROC, Informedness, Markedness & Correlation.” *J Mach Learn Technol* 2011; 2(1): 37-63.

Supplementary figure 1: mutual information formula

$$I(X;Y) = \sum_{x,y} p(x,y) \log \frac{p(x,y)}{p(x)p(y)}$$

This can also be thought of as the difference between the entropy of Y (i.e., H(Y)), and the entropy of Y given X (i.e., H(Y|X), the conditional entropy), where entropy can be interpreted as a measure of uncertainty. We used this metric to quantify the importance of each feature to outcome features.

Supplementary figure 2: Confusion matrices for A: direct head contact to the ball - carrier, B: direct head contact to the tackler, C: tackler head placement on contact. For all plots, the vertical axis shows the ground truth (human-determined) classification and the horizontal axis shows the classification determined by the best performing ML classification. The colour axis represents the number of tackle events in each category.

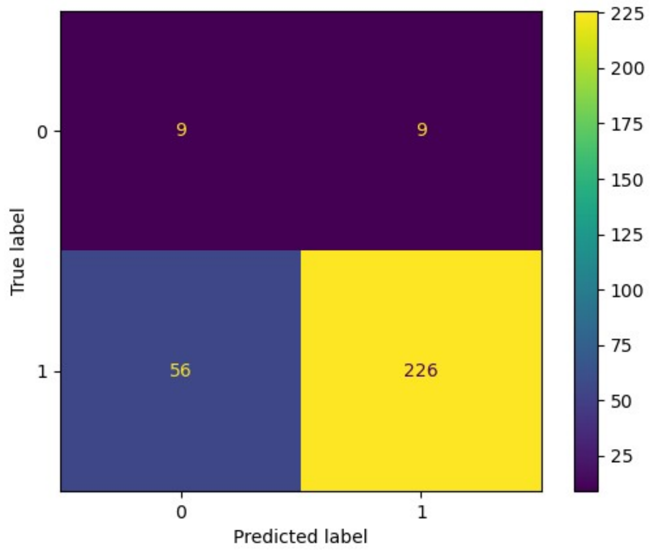

A: Confusion matrix for direct head contact to the ball-carrier. 0=direct head contact, 1=anywhere else

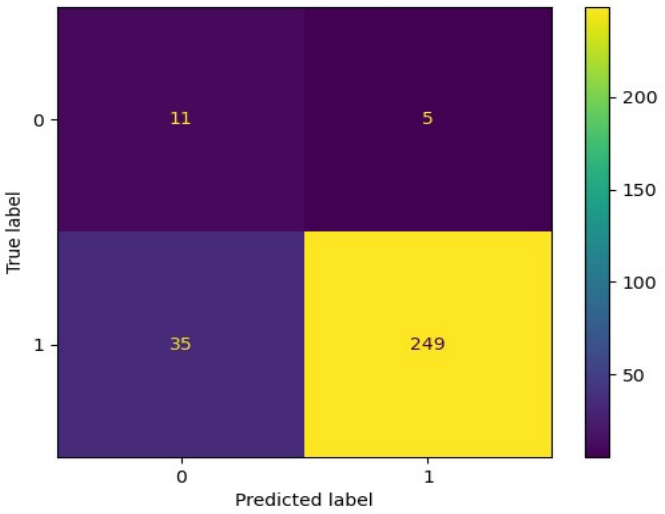

B: Confusion matrix for direct head contact to the tackler. 0=direct head contact, 1=anywhere else

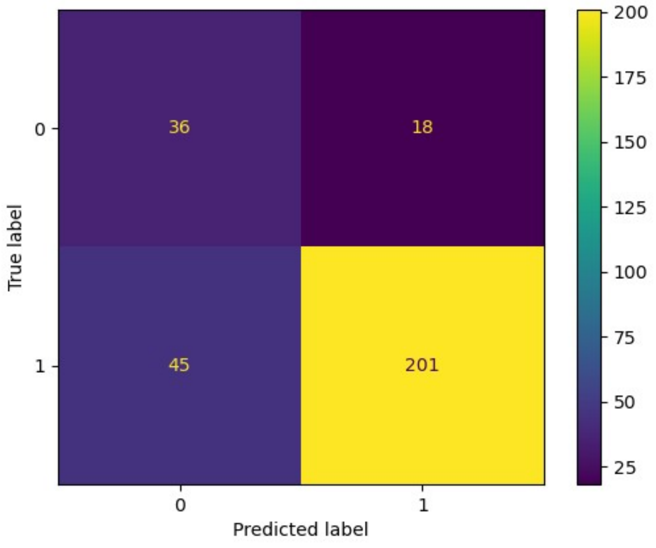

C: Confusion matrix for tackler head placement on contact. 0=incorrect tackler head placement, 1=correct tackler head placement.
